# FGCNSurv: dually fused graph convolutional network for multi-omics survival prediction

**DOI:** 10.1093/bioinformatics/btad472

**Published:** 2023-07-31

**Authors:** Gang Wen, Limin Li

**Affiliations:** School of Mathematics and Statistics, Xi’an Jiaotong University, Xi’an, Shaanxi 710049, China; School of Mathematics and Statistics, Xi’an Jiaotong University, Xi’an, Shaanxi 710049, China

## Abstract

**Motivation:**

Survival analysis is an important tool for modeling time-to-event data, e.g. to predict the survival time of patient after a cancer diagnosis or a certain treatment. While deep neural networks work well in standard prediction tasks, it is still unclear how to best utilize these deep models in survival analysis due to the difficulty of modeling right censored data, especially for multi-omics data. Although existing methods have shown the advantage of multi-omics integration in survival prediction, it remains challenging to extract complementary information from different omics and improve the prediction accuracy.

**Results:**

In this work, we propose a novel multi-omics deep survival prediction approach by dually fused graph convolutional network (GCN) named FGCNSurv. Our FGCNSurv is a complete generative model from multi-omics data to survival outcome of patients, including feature fusion by a factorized bilinear model, graph fusion of multiple graphs, higher-level feature extraction by GCN and survival prediction by a Cox proportional hazard model. The factorized bilinear model enables to capture cross-omics features and quantify complex relations from multi-omics data. By fusing single-omics features and the cross-omics features, and simultaneously fusing multiple graphs from different omics, GCN with the generated dually fused graph could capture higher-level features for computing the survival loss in the Cox-PH model. Comprehensive experimental results on real-world datasets with gene expression and microRNA expression data show that the proposed FGCNSurv method outperforms existing survival prediction methods, and imply its ability to extract complementary information for survival prediction from multi-omics data.

**Availability and implementation:**

The codes are freely available at https://github.com/LiminLi-xjtu/FGCNSurv.

## 1 Introduction

Survival analysis for cancer patients, which aims to model the relationship between multiple explanatory variables and survival outcome, has been an interesting and challenging problem in cancer research. With advances in high-throughput sequencing technologies, a great amount of high-dimensional genomic data is available for survival prediction and treatment improvement, and thus it has been promising to understand disease prognosis with these omics data with high dimensionality. Although the prediction of survival outcome resembles logistic regression in machine learning, the main difficulty is that the time-to-event data are often highly skewed and censored (Dey *et al.* 2022), where the former means a few patients may survive much longer than the median, and the latter means the failure to fully observe the time-to-event value for all patients due to the termination of the study, or missing patients during the study.

Survival analysis methods can be roughly divided into two categories: parametric models (Klein and Moeschberger 2003, Lemeshow *et al.* 2011) and Cox-based semi-parametric models (Cox 1972, Bradburn *et al.* 2003). The parametric models assume that the survival time of cancer patient obeys a specific distribution. The Cox proportional hazard (Cox-PH) model makes parametric assumptions about how predictors affect the hazard function, but has no restrictions on the baseline hazard function. Compared with parametric models, the Cox-PH model cannot estimate the hazard rate at each time point but can analyse the effect of covariates on survival (Kleinbaum and Klein 2012). Because the true hazard function is too complex to model in real world scenarios, the Cox-PH model becomes the most popular method. Furthermore, various extensions to Cox-PH model such as regularized Cox models (Park and Hastie 2007, Goeman 2010, Yang and Zou 2013) and deep neural networks supervised by Cox partial likelihood loss (Luck *et al.* 2017, Katzman *et al.* 2018, Kvamme *et al.* 2019) have been successfully proposed and showed promising performance.

Deep learning approaches have been widely used in survival analysis on a single type of omics data such as microRNA expression, gene expression, DNA methylation for survival prediction, basically by feature-based methods and graph-based methods. For example, Yousefi *et al.* (2017) proposed to use stacked denoising autoencoders to learn a low dimension feature representation of gene expression data for survival analysis. Ching *et al.* (2018) developed a Cox-nnet algorithm to predict prognosis of cancer patients. Cox-nnet uses hidden node features for dimension reduction and reveals much richer biological information at the gene levels by evaluating the relative importance of specific genes to the prognosis results. Since deep-learning methods based on limited high-dimensional gene expression data are prone to suffer from overfitting issues, Kim *et al.* (2020) put forward a VAECox algorithm, which pretrained a variational autoencoder on all gene expression data from TCGA and transferred the trained weights to survival prediction model of target cancer to reduce the possibility of overfitting. Recently, graph convolutional network (GCN), which exploits both node attributes and edges information in the form of a graph, has achieved promising performance in many areas including survival prediction. For example, Ling *et al.* (2022) proposed a survival model namely AGGSurv based on GCNs with geometric graphs directly constructed from high-dimensional RNA sequencing features. AGGSurv used random subsets of features to construct diverse sparse graphs and trained a Ridge-Cox model to learn how to best aggregate the predictions from the GCNs with the sparse graphs.

Multi-omics learning methods are considered to be more promising than single-omics methods since the integration of information from different sources are helpful to understand inter-patient heterogeneity and improve the performance of survival prediction (Chaudhary *et al.* 2018). According to the fusion strategies, the multi-omics methods can be categorized to feature-based fusion methods, which fuse the features from multiple omics, and graph-based fusion methods, which fuse the connections among samples from multiple omics. Feature fusion strategies enhance predictive performance of survival models by exploring intrinsic relationship of the features across different omics. For example, Cheerla and Gevaert (2019) developed an unsupervised encoder to compress patient multi-omics data into an informative representation for prognosis. Wang *et al.* (2021a) proposed GraphSurv method which introduced the KEGG pathway association relationship between genes in the form of a graph and used GCN to compress multi-omics data including mRNA, CNV and methylation to new embeddings for survival prediction. The GPDBN method (Wang *et al.* 2021b) and HFBSurv method (Li *et al.* 2022) integrated low-dimensional single-omics features by utilizing a bilinear feature encoding module to fully exploit intrinsic inter-modality and intra-modality relationships, and thus could improve the prediction performance. Besides feature fusion, graph-based fusion method GCGCN (Wang *et al.* 2020) improved the predictive performance of survival models by using SNF algorithm (Wang *et al.* 2014) to fuse multiple graphs for capturing shared and complementary information among multi-omics data. Although the advantages of multi-omics methods have been shown for survival prediction, it has large space to improve the prediction accuracy. For example, existing feature-based fusion methods may ignore the connections among different samples, while graph-based fusion methods are prone to lose the interactive information between features from multiple omics. It is still challenging to explore how to best utilize deep learning models for multi-omics survival prediction.

In this work, to take full advantages of the complementary information between different omics, we propose a novel multi-omics method for survival prediction namely FGCNSurv, by dually fusing features and graphs simultaneously in GCN. The generative model FGCNSurv learns a factorized bilinear model (FBM) to capture cross-omics features and quantify complex relations from multi-omics data. By fusing single-omics features and their cross-omics features (feature fusion), and simultaneously fusing multiple graphs from different omics (graph fusion), GCN with the generated dually fused graph could capture higher-level features for computing the survival loss in the Cox-PH model. To verify the effectiveness of FGCNSurv approach, we conducted comprehensive experiments on 11 cancer datasets from The Cancer Genome Atlas (TCGA) and the Pan-Cancer Analysis of Whole Genomes (PCAWG) dataset, and the results demonstrate that FGCNSurv shows better performance than other comparison methods for survival prediction.

## 2 Materials and methods

In this section, we first introduce and describe the problem of survival analysis, and then propose the dually fused GCN for multi-omics survival prediction.

### 2.1 Survival analysis

Survival data are usually given as ${\left\{x_{i},O_{i},\Delta_{i}\right\}}_{i=1}^{n}$, where $x_{i}$, *O_i_*, and Δ_*i*_ represent the observed features, the observed time-to-event and the censoring indicator for *i*th patient. Censoring occurs when a patient is lost or the maximum follow-up time is shorter than the survival time. For the *i*th patient, the observed time-to-event $O_{i}=\min(T_{i},C_{i})$ is either the true survival time *T_i_* for uncensored patients ($\Delta_{i}=1$), or the censoring time *C_i_*, for censored patient ($\Delta_{i}=0$).

The primary objective of survival analysis is to estimate the distribution of survival time *T* after diagnosis or treatment of a patient. The probability distribution of the survival time *T* can be characterized by a survival function or a hazard function equivalently. The survival function *S*(*t*) represents the probability of an individual surviving beyond time *t*: where *f*(*s*) represents the probability density function. The hazard function, denoted by λ(t), is defined as the rate that an event occurs in an infinitesimal interval after time *t*, given it has not yet occurred at time *t*:


$$S(t)=P(T>t)=1-\int_{0}^{t}f(s)ds,$$

$$\lambda (t)=\underset{\delta \to 0}{\lim}\frac{P(t\leq T<t+\delta |T>t)}{\delta }.$$

The full likelihood accounting for both censored and uncensored instances is


$$L=\prod_{i}f{\left(O_{i}|x_{i}\right)}^{\Delta_{i}}S{\left(O_{i}|x_{i}\right)}^{1-\Delta_{i}}$$

The Cox model is widely used in the survival analysis (Cox 1972) and assumes hazard function has a multiplicative form $\lambda (t|x_{i})=\lambda_{0}(t)\exp(\beta^{T}x_{i})$.

As the baseline hazard function $\lambda_{0}(\cdot )$ can be an arbitrary nonnegative function of time, one cannot estimate $\lambda_{0}(t)$ and *β* directly from the likelihood function. To deal with this estimation problem, the partial likelihood *L*_cox_ (Cox 1972) that does not involve the baseline hazard function is proposed to fit the model,


(1) $$L_{\mathrm{cox}}=\prod_{i}{\left(\frac{\exp(\beta^{T}x_{i})}{\sum_{j:O_{j}>O_{i}}exp(\beta^{T}x_{j})}\right)}^{\Delta_{i}}.$$

In practice, the negative partial log likelihood is often taken as loss function to estimate *β*:


(2) $$-\log(L_{\mathrm{cox}})=-\sum_{i=1}^{n}\Delta_{i}[\beta^{T}x_{i}-\log\sum_{j:O_{j}>O_{i}}\exp(\beta^{T}x_{j})].$$

With the estimated *β*, the hazard function λ(t) can be further estimated through the Breslow estimator (Darroch 1984).

### 2.2 Dually fused GCN for multi-omics survival prediction (FGCNSurv)

Multi-omics survival prediction aims to learn a survival prediction model based on multi-omics data for *n* patients and their corresponding observed times and right censoring indicators. In this work, each patient has both gene expression and microRNA expression representations, which are collected as $X^{\left(1\right)}=[x_{1}^{\left(1\right)},\ldots ,x_{n}^{\left(1\right)}]$ and $X^{\left(2\right)}=[x_{1}^{\left(2\right)},\ldots ,x_{n}^{\left(2\right)}]$, respectively, and thus the survival data are collected in set $D={\left\{x_{i}^{\left(1\right)},x_{i}^{\left(2\right)},O_{i},\Delta_{i}\right\}}_{i=1}^{n}$.

**Figure 1.**
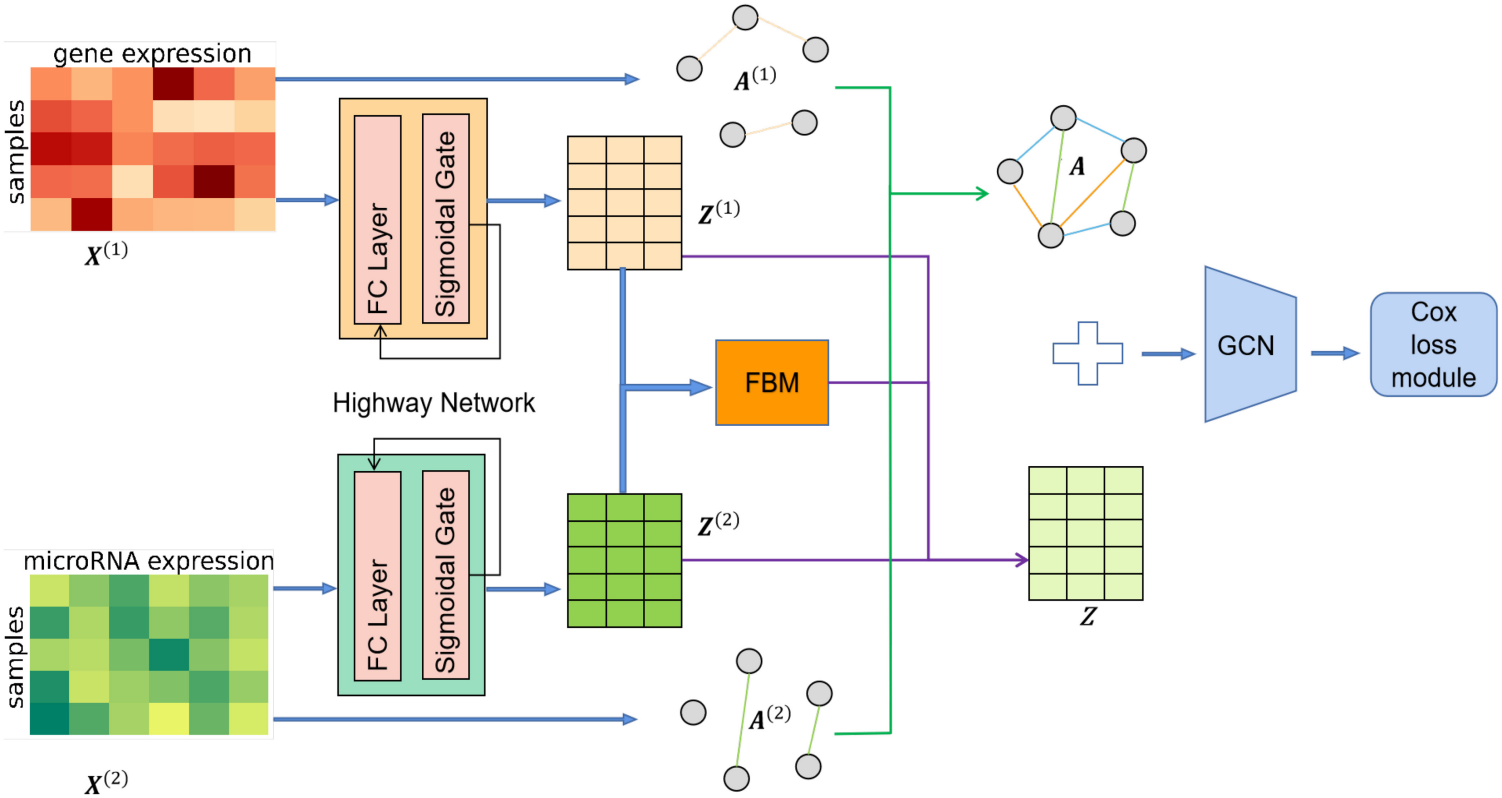
Illustration of the proposed FGCNSurv architecture. FGCNSurv first captures single-omics features by learnable highway networks, and further construct cross-omics features by a learnable FBM model. By fusing single-omics features and cross-omics features, and simultaneously fusing two single-omics graphs, a GCN model is used to further capture high-level features, which are then used to predict survival outcome by Cox-PH model

Our FGCNSurv takes a feature fusion strategy based on the FBM, which can capture cross-omics features and quantify complex relations from multi-omics data, and simultaneously fuses multiple graphs from different omics to accurately unveil the neighborhood relationship among patients. Feature fusion tends to aggregate complementary omics information for each patient, while graph fusion tends to share and propagate the complementary neighborhood information among patients from different omics. With the dually fused attributed graph, the GCN could further capture higher-level features for patients to better interchange the complementary information between the fused graph and features for survival prediction. The survival outcome is finally predicted from the node embeddings by GCN. The architecture of our proposed FGCNSurv is presented in Fig. 1. We describe the details for FGCNSurv in the following.

#### 2.2.1 Feature encoding by highway networks

For preprocessed gene expression data $X^{\left(1\right)}$ and microRNA expression data $X^{\left(2\right)}$, we learn two three-layer highway networks (Srivastava *et al.* 2015) to capture features, respectively. The highway network makes use of a LSTM-style sigmoidal gate to control gradient flow between fully connected (FC) layers, as shown below: where *H* represents an FC layer and *T* represents a transformation gate with sigmoid activation function. The highway network is commonly used in deep neural networks and simple tasks, to speed up information propagation by skipping layers that do not work well. Accordingly, we could obtain low-dimensional feature representations $Z^{\left(1\right)},\;Z^{\left(2\right)}\in R^{n\times l}$ for all patients with gene expression data and microRNA expression data, respectively.


$$x_{\mathrm{out}}=H(x_{\mathrm{in}})*T(x_{\mathrm{in}})+x_{\mathrm{in}}*(1-T(x_{\mathrm{in}})),$$

#### 2.2.2 Feature fusion by FBM

Multi-omics fusion for mining complementary information has been successfully introduced in cancer prognosis. Direct feature combination (Huang *et al.* 2019) or score level fusion (Sahasrabudhe *et al.* 2021) to integrate data from different omics may not be able to capture the complex inter-omics relations. To address this issue, we employ an FBM to capture cross-omics feature $z^{\left(c\right)}$ for each patient from its corresponding single-omics feature $z^{\left(1\right)}$ and $z^{\left(2\right)}$. Through the FBM, for any patient, the *j*th element in its cross-omics representation $z^{\left(c\right)}$ is defined as follows: where $U_{j}$ and $V_{j}$ are two factorized matrices with *d* columns. To obtain the cross-omics vector $Z^{c}\in R^{n\times m}$ for all patients, the FBM needs to learn two three-order tensors $U=[U_{1},\ldots ,U_{m}]\in R^{l\times d\times m}$ and $V=[V_{1},\ldots ,V_{m}]\in R^{l\times d\times m}$. The fused feature **Z** for all patients is finally obtained by concatenating the low level fusion feature $Z_{\mathrm{low}}=Z^{\left(1\right)}+Z^{\left(2\right)}$, which is the simple sum of omics-specific features, and the cross-omics feature $Z^{\left(c\right)}$, as defined below: where ⊕ represents the concatenation of two matrices.


$$z^{\left(c\right)}(j)=z^{\left(1\right)}{}^{T}U_{j}V_{j}^{T}z^{\left(2\right)},$$

(3) $$Z=Z_{\mathrm{low}}\;⊕\;Z^{\left(c\right)},$$

#### 2.2.3 Graph fusion

Although feature fusion could capture the comprehensive information from different omics for each single patient, it ignores the relationship among different patients. Our FGCNSurv further fuses multiple graphs constructed from single-omics data, and generates a fused graph which can accurately unveil the neighborhood relationship among patients. Specifically, given data representations ${\left\{x_{i}\right\}}_{i=1}^{n}$ for *n* patients, we denote $\rho (x_{i},x_{j})$ as the Euclidean distance between $x_{i}$ and $x_{j}$ and use the exponential similarity kernel to construct a *k*-nn graph with an adjacency matrix $A\in R^{n\times n}$ as follows: where *N_i_* represents *k*-nearest neighborhood for patient *i*, $\delta^{2}$ is used to eliminate the scaling problem, which is experimentally set to be the median of all pairwise squared distances among all patients and *μ* is a hyperparameter that can be set to 0.3 for gene-omics graph and 0.2 for microRNA-omics graph.


(4) $$A(i,j)=\{\begin{array}{cc} \;\text{exp}(\frac{-\rho^{2}(x_{i},x_{j})}{\mu \delta^{2}}), & j\in N_{i}\\ 0, & \text{otherwise}, \end{array}$$

By the above *k*-nn graph construction strategy, based on gene expression data $X^{\left(1\right)}$ and microRNA expression data $X^{\left(2\right)}$ for all patients, we could construct two single-omics graphs $A^{\left(1\right)}$ and $A^{\left(2\right)}$, respectively. Then we fuse the two graphs by taking the average of the two adjacent matrices $A^{\left(1\right)}$ and $A^{\left(2\right)}$, to obtain a fused graph with adjacent matrix **A** for survival analysis.

#### 2.2.4 Dually fused GCN for survival prediction

Graph convolutional neural network (GCN) could aggregate and exchange information between neighboring nodes (samples) to obtain embedding for nodes of graph, and thus has been applied successfully in many classification tasks (Kipf and Welling 2016, Veličković *et al.* 2017, Gao and Ji 2019). To capture higher-level features for survival prediction, we could further combine the multi-omics fused feature representations **Z** and the fused graph **A** by GCN. Given graph **A**, the convolution matrix $\hat{A}$ in GCN is given by ${\tilde{D}}^{-\frac{1}{2}}\tilde{A}{\tilde{D}}^{-\frac{1}{2}}$, where $\tilde{A}=A+I_{n}$ and $\tilde{D}=\mathrm{diag}({\tilde{d}}_{1},{\tilde{d}}_{2},\ldots ,{\tilde{d}}_{n})$ is the degree matrix calculated from $\tilde{A}$ with ${\tilde{d}}_{i}=\sum_{j}{\tilde{A}}_{ij}$. With node representation matrix $Z=[z_{1},z_{2},\ldots ,z_{n}]$ for *n* patients and convolution matrix $\hat{A}$, we could adapt the GCN to learn the higher-level features $Z^{h}$ as shown below: where $W_{1},W_{2}$ are the weight matrices for the graph convolutional layers and *f*_1_, *f*_2_ are tanh and sigmoid activation functions, respectively. Based on the features $z_{i}^{h}$, the Cox model $\lambda (t|z_{i}^{h})=\lambda_{0}(t)\exp(\beta^{T}z_{i}^{h})$ is used to perform survival prediction with parameter *β*. The loss function of the FGCNSurv model is defined as the negative of the partial loglikelihood: and all the parameters are learnt by minimizing the loss function.


(5) $$Z^{h}=f_{2}(f_{1}(\hat{A}ZW_{1})W_{2}),$$

(6) $$\mathrm{Loss}=-\sum_{i=1}^{n}\Delta_{i}[\beta^{T}z_{i}^{h}-\log\sum_{j:O_{j}>O_{i}}\exp(\beta^{T}z_{j}^{h})],$$

To learn a FGCNSurv model based on multi-omics survival data, we need to learn four modules including highway networks for capturing single-omics features, FBM for further capturing cross-omics features, GCNs for capturing high-level features based on the constructed dually fused graph, and finally Cox-PH model for predicting the survival outcome.

## 3 Results

In this section, we evaluated the survival prediction performance of our FGCNSurv method by comparing it with other methods on 11 experiments from two datasets, using different evaluations metrics.

### 3.1 Datasets and preprocessing

The comparison of survival analysis performance was conducted on 11 different cancer datasets from The Cancer Genome Atlas (TCGA) and the Pan-Cancer Analysis of Whole Genomes (PCAWG) dataset as follows:

- TCGA dataset (Zhu *et al.* 2014) TCGA dataset includes multi-omics data for over 11 000 samples from 33 most frequent cancer types and has been widely used in survival prediction methods (Sun *et al.* 2018a, Shao *et al.* 2020, Tong *et al.* 2020). We selected top 10 cancer types with respect to the number of uncensored patient, except LUSC and STAD due to the poor performance of single-omics methods on both gene expression and microRNA expression. The 10 selected cancer types from TCGA are listed as follows: breast invasive carcinoma (BRCA), kidney renal clear cell carcinoma (KIRC), lung adenocarcinoma (LUAD), urothelial bladder carcinoma (BLCA), head and neck squamous cell carcinoma (HNSC), brain lower grade glioma (LGG), liver hepatocellular carcinoma (LIHC), ovarian serous cystadenocarcinoma (OV), colon adenocarcinoma (COAD), and skin cutaneous melanoma (SKCM). We downloaded RNA-Seq data for gene expression and miRNA-Seq data for microRNA expression to evaluate the multi-omics survival prediction methods.
- PCAWG dataset (Campbell *et al.* 2018), the Pan-Cancer Analysis of Whole Genomes (PCAWG) Consortium aggregated whole-genome sequencing data from 2658 cancer patients across 38 tumor types generated by the International Cancer Genome Consortium (ICGC) and The Cancer Genome Atlas (TCGA) projects. We also downloaded RNA-Seq data for gene expression and miRNA-Seq data for microRNA expression to evaluate the multi-omics survival prediction methods.

The summary of the 11 datasets were listed in Table 1. We preprocessed each dataset with gene expression and microRNA expression data using the following procedure. First, similar to prior work (Dhillon and Singh 2020), we removed the genes/microRNAs with missing values over 10%, estimated the missing values by weighted nearest neighbors algorithm and added a pseudocount 1 to all features, followed by a log transformation. Second, we filtered out the low information burden genes/microRNAs whose values remain almost unchanged across samples to reduce the number of noise-sensitive features, by which the top 6000 genes and 600 microRNA features are chosen, respectively. Finally, we standardized the log-transformed data such that each feature has zero mean and unit variance, and then discretized them to multiple categories.

**Table 1.** Summary of the datasets used in the experiments.

Dataset	Patients	Prop. censored	Dataset	Patients	Prop. censored
BRCA	1021	0.861	OV	372	0.390
KIRC	508	0.671	COAD	439	0.770
HNSC	495	0.564	LGG	504	0.754
LUAD	496	0.637	LIHC	367	0.651
BLCA	402	0.562	SKCM	437	0.515
PCAWG	832	0.755			

### 3.2 Evaluation metrics

In this study, we evaluated the performance of FGCNSurv in survival prediction with two metrics: C-index and AUC. The C-index is the most commonly used evaluation metric to measure the predictive ability of the model in survival analysis, which estimates the probability that the predicted results are consistent with the observed ground truth results. For any pair of patients, if the predicted survival time of the one with longer survival time is also longer than the predicted survival time of the other, the predicted outcome is consistent with the actual outcome. A pair is not considered if the earlier time in the pair is censored or both events in the pair are censored. The C-index is defined by:


(7) $$C-\text{index}=\mathrm{Prob}\{\beta^{T}z_{i}^{h}>\beta^{T}z_{j}^{h}|O_{j}>O_{i},\Delta_{i}=1\},$$

Another evaluation metric, AUC, quantifies the quality of the ranking at event-time level and is defined as follows: where *Y* and num refer to the set of all possible event time duration in the dataset and the cumulative number of comparable pairs computed across all event time duration, respectively, and I(·) is the indicator function. Both the values of C-index and AUC range from 0 to 1, and a higher value of C-index or AUC indicates better model prediction performance and vice versa.


(8) $$\text{AUC}=\frac{1}{\mathrm{num}}\sum_{t\in Y}\sum_{i:O_{i}<t}\sum_{j:O_{j}>t}I(\beta^{T}z_{i}^{h}>\beta^{T}z_{j}^{h})$$

### 3.3 Evaluation of survival prediction performance

In our experiments, we compared our FGCNSurv method with existing traditional and deep learning-based survival prediction methods on 11 cancer datasets obtained from TCGA and PCAWG dataset. The comparison methods consist of five single-omics methods including RSF (Ishwaran *et al.* 2008) and En-Cox (Yang and Zou 2013), DeepSurv (Katzman *et al.* 2018), DeepHit (Lee *et al.* 2018), AGGSurv (Ling *et al.* 2022), and three multi-omics methods including score fusion method MDNNMD (Sun *et al.* 2018b), graph fusion method GCGCN (Wang *et al.* 2020), and bilinear product-based method HFBSurv (Li *et al.* 2022). All the methods except RSF and En-Cox are deep learning methods.

To comprehensively evaluate our survival prediction method, we adopt repeated holdout cross-validation following Ching *et al.* (2018). In particular, the dataset was randomly partitioned into 80% training set and 20% testing set. We trained the prediction models by different methods on training set, and then calculated the C-index and AUC by survival prediction for the test patients. We repeated the process for 20 times and reported the mean value and standard deviation of the C-indices and AUCs for each method. For our method FGCNSurv, the dimensions *l* and *m*, i.e. the dimensionality of the feature vector $Z_{low}$ of low-level fusion and the cross-omics features $Z^{\left(c\right)}$, were set to 50 and 10, respectively. The neighborhood size *k* for graph construction was selected by cross-validation on the training data from the set {2,…,15}. We trained the model with the stochastic gradient descent algorithm Adam optimizer with learning rate 2e−4.

We first reported the C-index and AUC values for the breast cancer dataset BRCA from TCGA by different prediction methods in Table 2. Deep learning-based approaches could generally obtain better performance than traditional methods, by only using single-omics data. For example, the deep single-omics method DeepSurv could obtained much higher C-index value than the traditional single-omics method En-Cox, with significant improvement on gene expression and microRNA expression data, respectively. Another deep single-omics method AGGSurv tends to achieve more competitive performance than DeepSurv for either omics, probably because the graph embedding used in AGGSurv could exploit the connections among the samples. More importantly, the multi-omics prediction methods including GCGCN, HFBSurv and our FGCNSurv perform better than single-omics methods, with respect to both C-index and AUC values. Our FGCNSurv could obtain the highest C-index and AUC value among all the methods. These results show the advantages of the FGCNSurv and imply that it could deeply capture the complementary information from multi-omics data for survival prediction.

**Table 2.** Performance comparison of FGCNSurv and existing methods using C-index and AUC values on BRCA dataset.

Data	Method	C-index	AUC
Gene/microRNA	RSF	0.578/0.594	0.594/0.621
	En-Cox	0.593/0.603	0.617/0.628
	DeepSurv	0.693/0.652	0.726/0.678
	DeepHit	0.687/0.632	0.718/0.656
	AGGSurv	0.701/0.654	0.733/0.679
Gene + microRNA	MDNNMD	0.680 ± 0.061	0.712 ± 0.057
	GCGCN	0.716 ± 0.046	0.751 ± 0.049
	HFBSurv	0.723 ± 0.039	0.756 ± 0.051
	FGCNSurv	**0.740 ± 0.043**	**0.773 ± 0.050**

The best results are shown in bold.

We also reported the prediction results for the other 10 experiments, including nine TCGA cancer datasets and the PCAWG dataset, with the C-indices in Table 3. As can be seen from the table, our method outperformed existing methods on the 7 of 10 TCGA cancer datasets and the PCAWG dataset, and achieved the second-best C-index values for the other three TCGA cancer datasets. Furthermore, for LIHC dataset, FGCNSurv has a significant improvement over the second-best method. Overall, the results on the total 11 datasets show the advantage of FGCNSurv for predicting survival of different cancer patients with multi-omics data. Similarly to C-indices, FGCNSurv could also obtain better AUCs than other methods, which were skipped here due to the space limitation.

**Table 3.** Performance comparison of FGCNSurv and existing methods using C-index values on 9 TCGA datasets and PCAWG dataset.

		C-index									
Data	Method	KIRC	HNSC	LUAD	BLCA	LGG	COAD	SKCM	OV	LIHC	PCAWG
Single	RSF	0.671/0.624	0.598/0.607	0.585/0.602	0.622/0.608	0.816/0.652	0.594/0.577	0.616/0.628	0.566/0.591	0.656/0.613	0.617/0.652
	En-Cox	0.606/0.593	0.616/0.602	0.564/0.572	0.619/0.603	0.831/0.688	0.613/0.586	0.589/0.596	0.582/0.575	0.628/0.607	0.605/0.627
	DeepSurv	0.731/0.687	0.630/0.615	0.627/0.616	0.666/0.661	0.840/0.795	0.632/0.591	0.653/0.630	0.601/0.594	0.691/0.660	0.724/0.727
	DeepHit	0.729/0.678	0.634/0.608	0.629/0.626	0.657/0.651	0.836/0.819	**0.675**/0.626	0.652/0.626	0.616/0.569	0.685/0.625	0.726/0.717
	AGGSurv	0.726/0.695	0.635/0.619	0.621/0.628	0.664/0.663	0.849/0.805	0.647/0.602	0.648/0.625	0.606/0.593	0.693/0.667	0.728/0.731
Multi	MDNNMD	0.709	0.633	**0.654**	0.637	0.834	0.655	0.642	**0.639**	0.682	0.718
	GCGCN	0.736	0.622	0.640	0.667	0.856	0.656	0.658	0.603	0.689	0.738
	HFBSurv	0.721	0.632	0.639	0.662	0.844	0.658	0.643	0.624	0.665	0.697
	FGCNSurv	**0.745**	**0.641**	0.651	**0.674**	**0.860**	0.670	**0.660**	0.626	**0.703**	**0.746**

The best and second best results are highlighted in bold and underlined respectively.

**Figure 2.**
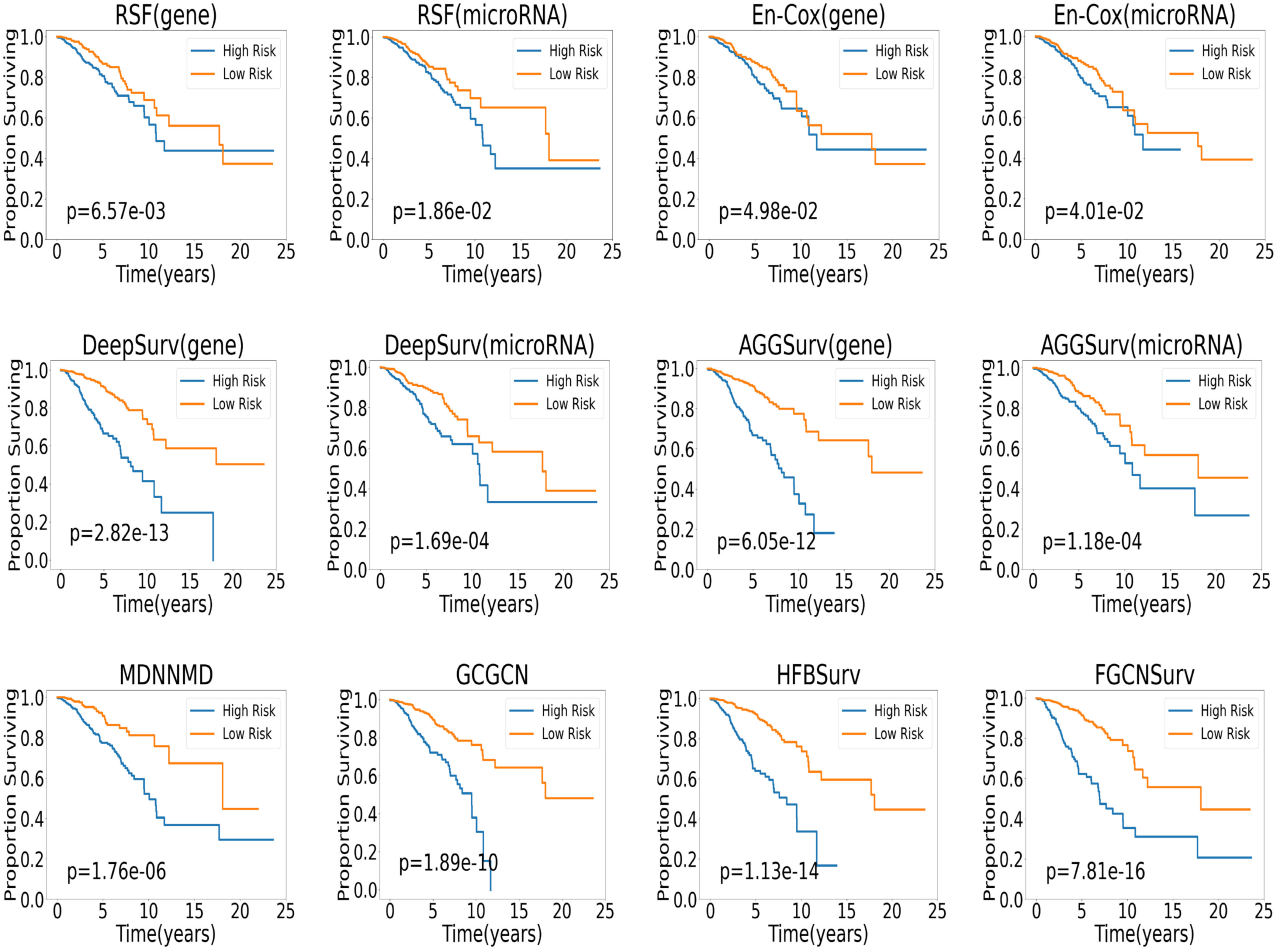
Kaplan–Meier curves of predicted high risk group and low risk group obtained by different single-omics survival prediction methods (first two rows) and multi-omics survival prediction methods (bottom row) on BRCA dataset

To further evaluate the performance of FGCNSurv, we performed the logrank test to test whether the patients in predicted high risk group and predicted low risk group have significantly different survival curves. Specifically, we divided the patients in the BRCA dataset into a high risk group and a low risk group based on their predicted hazard ratios, by using the threshold of the median hazard ratios. We then conducted the log-rank test to test whether the ground truth survival time of the samples in the two group are significantly different. The more significant is the difference between the two groups, the better is the prediction method. In Fig. 2, we plotted the Kaplan–Meier curves for the predicted high risk group and low risk group obtained by all methods based on the breast cancer dataset, and also reported the log-rank *P*-values. We can see that, for single-omic methods, deep learning method DeepSurv could obtain better results than traditional methods En-Cox and RSF for either omics. For example, DeepSurv obtained more significant log-rank *P*-values of 2.82e−13 and 1.69e−04 than En-Cox (4.98e−02 and 4.01e−02) and RSF (6.57e−03 and 1.86e−02) for gene expression and microRNA expression, respectively. This implies that DeepSurv could produce better survival predictions with more significant differences between the high risk group and low risk group. Furthermore, multi-omics methods including HFBSurv and our FGCNSurv could obtain even more significant results than the above two deep learning based single-omics methods DeepSurv and AGGSurv. Furthermore, our proposed FGCNSurv generated the most significant *P*-value of 7.81e−16, among all the four muti-omics methods, and this shows that our FGCNSurv could obtain the best survival prediction with more significant differences between the high risk group and low risk group, which reflects the effectiveness of the proposed method in survival prediction.

### 3.4 Univariate and multivariate Cox proportional hazards analysis

To evaluate whether the survival prediction performance of FGCNSurv is associated with five clinicopathologic factors of patients, including age at diagnosis, histologic grade, tumor size (T stage), lymph node invasion (N stage), and metastatic spread (M stage), we classified the BRCA patients to a high risk group and a low risk group by the predicted risks from the test sets, and then took the predicted risk as the sixth factor besides the above five. We then performed univariate and multivariate Cox proportional hazards analysis on the six factors including our predicted risk and clinicopathologic factors (Yu *et al.* 2019). Table 4 reports the hazard ratio by using different factors in univariate and multivariate analysis. As shown in Table 4, two factors of histologic grade and the predicted risk show significant association with survival in both univariate and multivariate Cox proportional analysis, and our predicted risk shows the most evident hazard for survival among all the six factors. The multivariate analysis implies that our risk factor could retain strong and significant independent prognostic factor when correcting for other clinicopathologic variables. The above analysis demonstrates that our FGCNSurv has great predictive power for survival.

**Table 4.** Hazard ratios for univariate and multivariate Cox proportional hazards analysis on BRCA dataset.

		Univariate			Multivariate		
Variable		Hazard ratio	95% CI	*P* value	Hazard ratio	95% CI	*P* value
Age	≤50/>50	1.50	1.00–2.26	.05	1.52	1.00–2.29	.05
Grade	≤II/>II	2.27	1.57–3.29	<.005	2.74	1.23–6.14	.01
T Stage	≤T2/>T2	1.60	1.05–2.44	.03	0.92	0.48–1.75	.79
N Stage	≤N1/>N1	2.06	1.34–3.17	<.005	0.91	0.43–1.94	.81
M Stage	M0/MX	0.70	0.32–1.51	.36	0.62	0.28–1.34	.22
FGCNSurv	Low risk/High risk	2.20	1.51–3.21	<.005	2.16	1.48–3.17	<.005

### 3.5 Ablation study and parameter robustness

To verify the contribution of our dual fusion strategy in FGCNSurv for multi-omics survival prediction, we conducted ablation experiments on BRCA dataset by changing different parts in our model configuration. We adopted the following six different configurations to evaluate each component of the proposed method:

- GCN: GCN on the single-omics graph with patient features obtained from highway networks.
- LOW-FF: only using low-level feature fusion, i.e. the simple sum of omics-specific features $Z^{\left(1\right)}$ and $Z^{\left(2\right)}$, for loss construction, without FBM and GCN modules.
- FBM-FF: only using the cross-omics features $Z^{\left(c\right)}$ by FBM for loss construction, without GCN module.
- HIGH-FF: using high-level feature fusion by concatenating the low-level features $Z_{low}$ and the cross-omics features $Z^{\left(c\right)}$, without GCN module.
- w/o Graph: removing the graph from the whole network structure, and replacing the graph by an identity matrix in GCN.
- w/o FBM: removing FBM module from the whole network structure, and replacing **Z** by the low-level features $Z_{low}$.

We performed the above six variants of FGCNSurv by changing different parts on the BRCA dataset. GCN is single-omics methods, LOW-FF, FBM-FF, and HIGH-FF are feature fusion methods using different fusion strategies without GCN, w/o graph is FGCNSurv without graph, i.e. the high-level feature fusion followed by a fully connected network, and w/o FBM is FGCNSurv without FBM, i.e. removing FBM from the FGCNSurv. For a fair comparison, we used the same neural networks in the above six configurations to extract the features of each omics.

We reported the C-index and AUC values for different configurations in ablation study of FGCNSurv for BRCA dataset in Table 5, and other datasets in Supplementary Table 1. We can see that from Table 5 that, even without GCN module, our feature fusion strategy HIGH improves the simple fusion LOW and FBM. It can be also seen that FGCNSurv outperforms w/o FBM with respect to both C-index and AUC values, which reflects the importance of cross-omics features in our dual fusion strategy. Furthermore, the C-index and AUC values of FGCNSurv both have improvement over w/o Graph, implying the effectiveness of our graph-fusion strategy. The ablation study shows that the graph fusion strategy and factorized bilinear module are two important factors for the performance improvement of FGCNSurv.

**Table 5.** C-index and AUC values for different configurations in ablation study of FGCNSurv on BRCA dataset.

Data	Method	C-index	AUC
Gene/microRNA	GCN	0.715/0.663	0.744/0.691
Gene + microRNA	LOW-FF	0.692 ± 0.064	0.719 ± 0.066
	FBM-FF	0.687 ± 0.075	0.711 ± 0.062
	HIGH-FF	0.706 ± 0.058	0.732 ± 0.064
	w/o Graph	0.728 ± 0.044	0.762 ± 0.051
	w/o FBM	0.732 ± 0.041	0.766 ± 0.040
	FGCNSurv	**0.740 ± 0.043**	**0.773 ± 0.050**

The best results are shown in bold.

**Figure 3.**
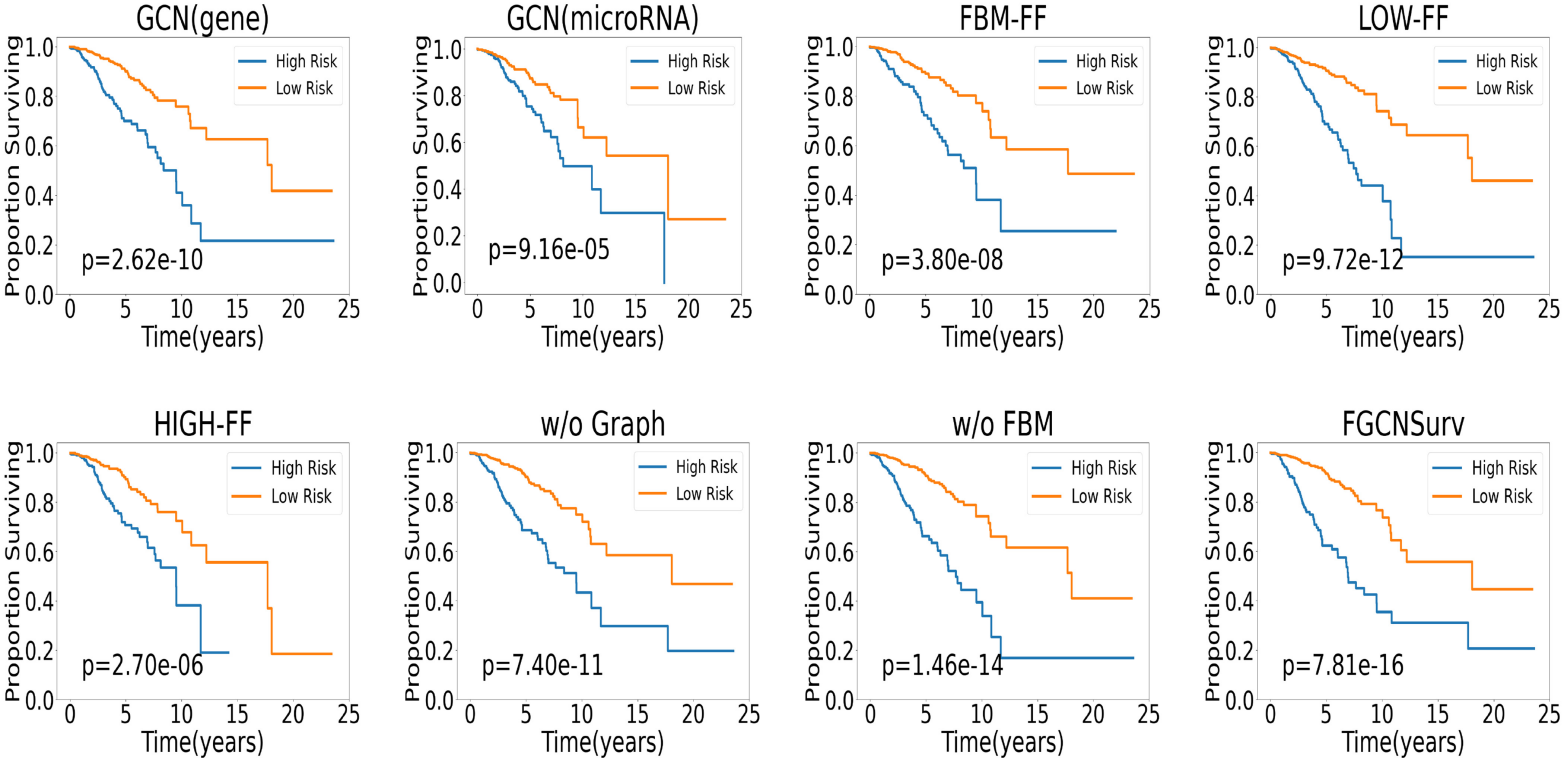
Kaplan–Meier curves of predicted high risk group and low risk group obtained by different configurations in ablation study of FGCNSurv on BRCA dataset

We also plotted the corresponding Kaplan–Meier curves of the predicted results by the above approaches in Fig. 3, similar to Section 3.3. We can see that FGCNSurv gives the most significant *P*-value of 7.81e−16 among all configurations in ablation experiments. The results show that FGCNSurv enables better classification of patients into low and high risk groups with remarkably better stratification, and imply the dual fusion strategy can explore the complementary information of microRNA and gene data for prognosis prediction of breast cancer.

**Figure 4.**
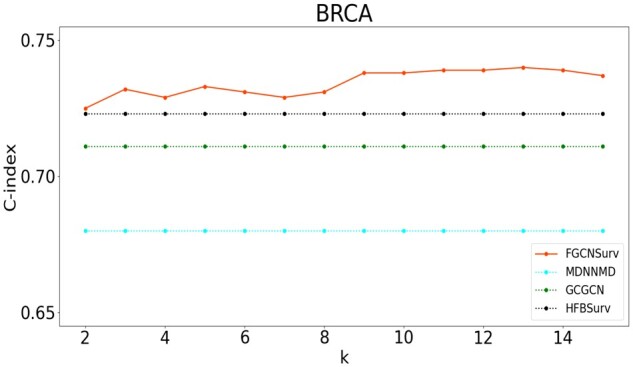
Performance of FGCNSurv under different values of hyper-parameter *k* on BRCA dataset. The dashed lines represent the results from the existing multi-omics integration methods

Although the only hyperparameter *k* in FGCNSurv, i.e. the neighborhood size for constructing *k*-nn graph, could be selected by cross-validation on training data, to show its robustness in a relatively large interval, we trained the FGCNSurv with different *k* values from 2 to 15, and reported the resulting C-indices for BRCA dataset by FGCNSurv in Fig. 4 and other datasets in Supplementary Fig. 1. We can see that, although the C-index obtained by FGCNSurv changes when *k* varies, it tends to be robust, especially when *k* is from 8 to 15, and furthermore, FGCNSurv could always obtain better C-indices than other comparison methods, with different *k*-values in a relatively large interval. This implies the robustness of the selection of *k* in real applications.

## 4 Conclusion

In this study, we proposed a novel cancer survival prediction method FGCNSurv by dually fused GCN. Different from the existing feature fusion or graph fusion methods for multi-omics survival prediction, FGCNSurv takes a strategy of both feature fusion and graph fusion to capture the complementary information across different omics. The experiments on 11 cancer datasets show its advantages for survival prediction. Although FGCNSurv has obtained the best predictive performance, survival prediction is still a challenging problem and there is large space to improve the accuracy. For example, the prediction accuracy might be improved by introducing other omics data such as DNA methylation or pathological images, which have been reported to be helpful for cancer prognosis prediction. Besides, proportional hazard model in our FGCNSurv approach assumes that the patient risk does not vary over time, which is too restrictive in real case. It is promising to further improve our framework by jointly predicting the time of event and its rank in the Cox partial log-likelihood framework based on multi-task learning.

## Supplementary Material

btad472_Supplementary_Data

## Data Availability

All data used in this manuscript are publicly available. The TCGA dataset used in this work can be obtained from the website https://www.cancer.gov/ccg/research/genome-sequencing/tcga. The PCAWG dataset is available on the website http://xena.ucsc.edu/.
