# Supplementary material for: FGCNSurv: dually fused graph convolutional network for multi-omics survival prediction
Source: Bioinformatics. 2023 Jul 31;39(8):btad472. doi: 10.1093/bioinformatics/btad472 (PMC10412406; doi:10.1093/bioinformatics/btad472)
Supplement: btad472_Supplementary_Data [file btad472_supplementary_data.pdf]

Supplementary Material

In this supplementary material, we first reported the results for ablation study on all the datasets in Table 1. In this table, we also showed the importance of highway networks by comparing w/o HW method, which replaces the 3-layer highway network to encode features with a two-layer fully connected network, with the FGCNSurv. The results show that, on the 7 of 10 TCGA cancer datasets and the PCAWG dataset, the FGCNSurv outperforms its seven variates, which implies the importance of each module in the method, including the highway network. Furthermore, these results clearly highlight the contribution of conducting graph fusion and feature fusion based on factorized bilinear model.

To show the robustness of the parameter k, i.e., the neighborhood size for constructing k-nn graph, in a relatively large interval, we trained the FGCNSurv with different k values from 2 to 15. We reported the resulting C-indices for all the datasets by FGCNSurv in Supplementary Figure 1, for TCGA datasets and PCAWG dataset, respectively. We can see that, although the C-index obtained by FGCNSurv changes when k varies, it tends to be robust when k is in a relatively large interval. This implies the robustness of the selection of k in real applications.

We also experimentally showed the convergence of the proposed method FGCNSurv on BRCA dataset. As can be seen from Figure 2, the loss of the method FGCNSurv on the training set gradually decreases with the number of iterations increasing.

Table 1: C-index values for different configurations in ablation study of FGCNSurv

	C-index							
Cancer	GCN	FBM-FF	LOW-FF	HIGH-FF	w/o Graph	w/o FBM	w/o HW	FGCNSurv
BRCA	0.715/0.663	0.687	0.692	0.706	0.728	0.732	0.730	**0.740**
KIRC	0.736/0.713	0.715	0.738	0.720	0.742	0.738	0.742	**0.745**
HNSC	0.629/0.595	0.616	0.628	0.633	0.636	0.638	0.637	**0.641**
BLCA	0.656/0.645	0.657	0.667	0.663	0.662	0.670	**0.677**	0.674
LGG	0.849/0.826	0.853	0.854	0.855	**0.860**	0.858	0.858	**0.860**
COAD	0.639/0.611	0.646	0.655	0.665	0.658	0.667	0.667	**0.670**
OV	0.612/0.576	0.603	0.617	0.622	0.614	0.620	0.618	**0.626**
LIHC	0.683/0.664	0.654	0.687	0.686	0.684	0.698	**0.708**	0.703
SKCM	0.657/0.650	0.651	0.656	0.646	0.652	0.656	0.656	**0.660**
LUAD	0.643/0.640	0.638	0.646	0.636	0.635	0.650	**0.655**	0.651
PCAWG	0.715/0.697	0.692	0.714	0.727	0.744	0.739	0.738	**0.746**

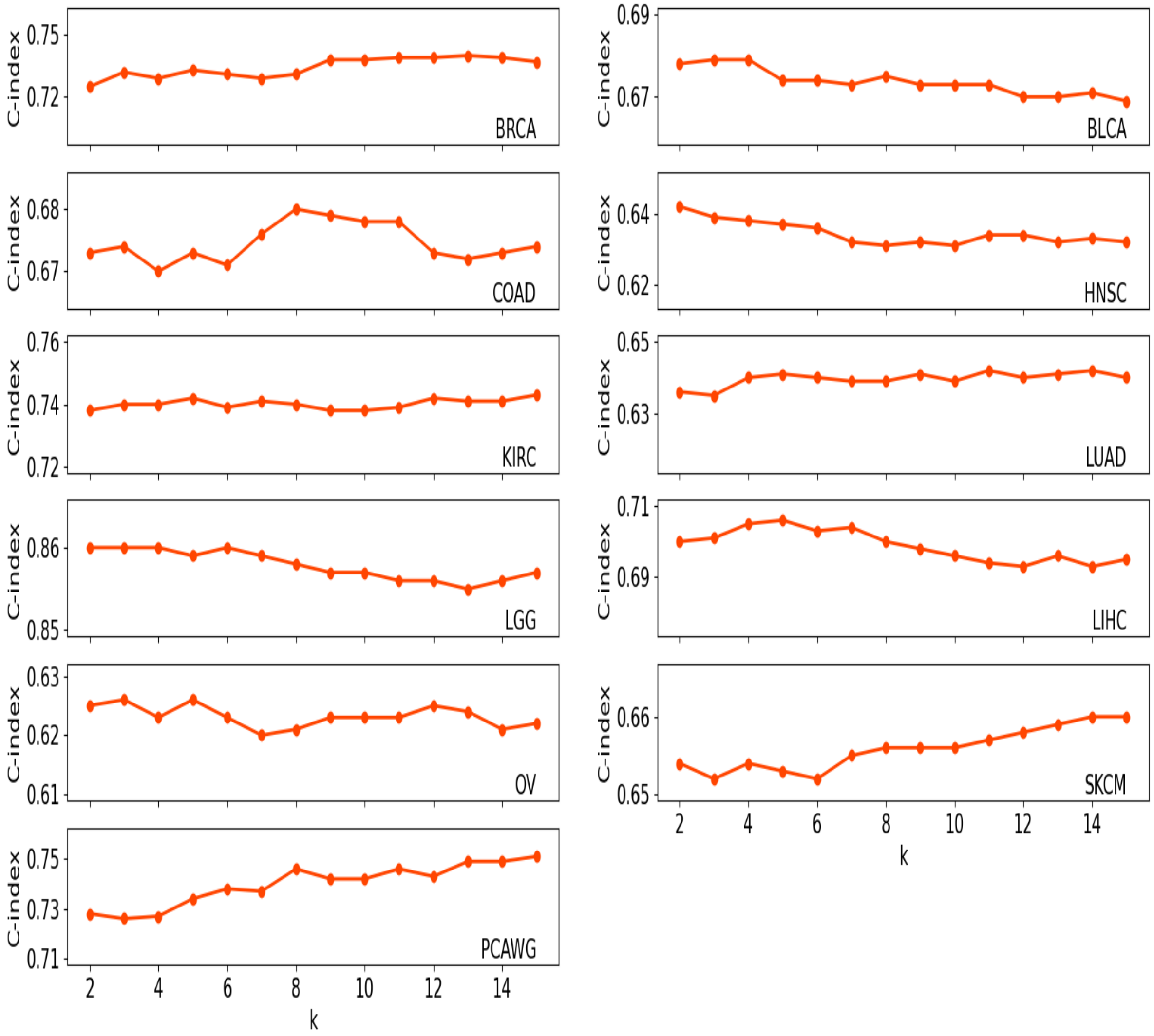

Figure 1: Performance of FGCNSurv under different values of hyper-parameter k on TCGA datasets and PCAWG dataset

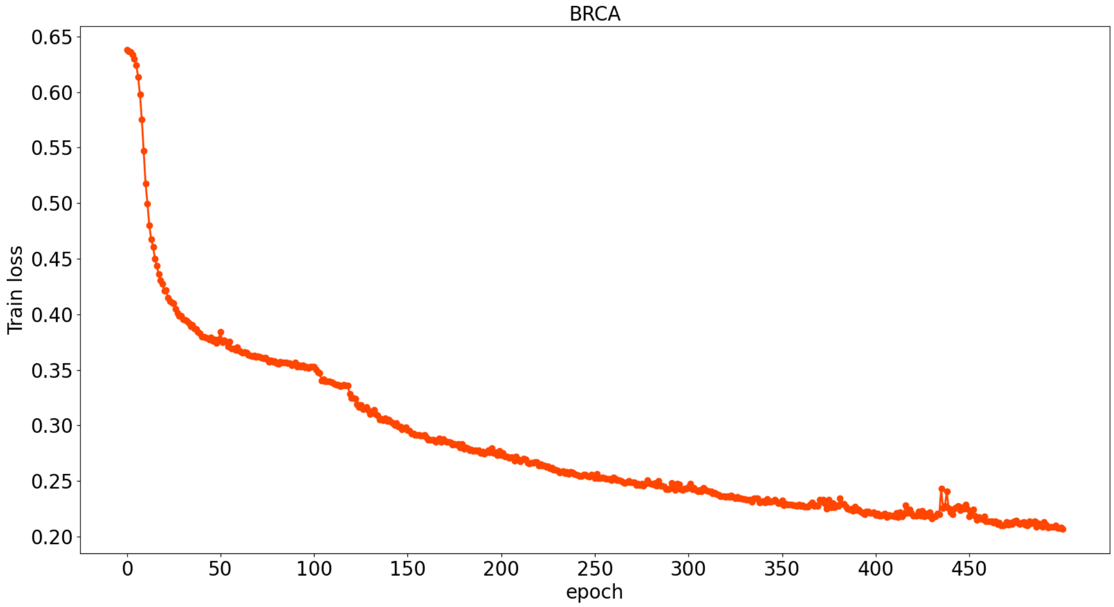

Figure 2: Convergence analysis of FGCNSurv method on BRCA dataset
